# Diagnostic Impact of Radiological Findings and Extracellular Vesicles: Are We Close to Radiovesicolomics?

**DOI:** 10.3390/biology10121265

**Published:** 2021-12-03

**Authors:** Francesco Lorenzo Serafini, Paola Lanuti, Andrea Delli Pizzi, Luca Procaccini, Michela Villani, Alessio Lino Taraschi, Luca Pascucci, Erica Mincuzzi, Jacopo Izzi, Piero Chiacchiaretta, Davide Buca, Giulia Catitti, Giuseppina Bologna, Pasquale Simeone, Damiana Pieragostino, Massimo Caulo

**Affiliations:** 1 Department of Neuroscience, Imaging and Clinical Sciences, University “G. d’Annunzio”, 66100 Chieti, Italy; francesco.serafini@unich.it (F.L.S.); luca.procaccini93@gmail.com (L.P.); michelavil92@gmail.com (M.V.); alessiotaraschi@aruba.it (A.L.T.); luca-pascucci@live.it (L.P.); erica.mincuzzi@gmail.com (E.M.); jacopoizzi@live.it (J.I.); p.chiacchiaretta@unich.it (P.C.); massimo.caulo@unich.it (M.C.); 2 Department of Medicine and Aging Sciences, University “G. d’Annunzio”, 66100 Chieti, Italy; p.lanuti@unich.it (P.L.); bucadavide@gmail.com (D.B.); catittig@gmail.com (G.C.); giuseppina.bologna@hotmail.it (G.B.); simeone.pasquale@gmail.com (P.S.); 3 Center for Advanced Studies and Technology (CAST), University “G. d’Annunzio”, 66100 Chieti, Italy; dpieragostino@unich.it; 4 Institute of Advanced Biomedical Technologies (ITAB), University “G. d’Annunzio”, 66100 Chieti, Italy; 5 Department of Innovative Technologies in Medicine & Dentistry, University “G. d’Annunzio”, 66100 Chieti, Italy

**Keywords:** extracellular vesicles, radiology, radiomics, artificial intelligence, radiovesicolomics

## Abstract

**Simple Summary:**

Over the years, diagnostic tests such as in radiology and flow cytometry have become more and more powerful in the constant struggle against different pathologies, some of which are life-threatening. The possibility of using these “weapons” in a conjugated manner could result in higher healing and prevention rates, and a decrease in late diagnosis diseases. Different correlations among pathologies, extracellular vesicles (EVs), and radiological findings were recently demonstrated by many authors. Together with the increasing importance of “omics” sciences, and artificial intelligence in this new century, the perspective of a new research field called “radiovesicolomics” could be the missing link, enabling a different approach to disease diagnosis and treatment.

**Abstract:**

Currently, several pathologies have corresponding and specific diagnostic and therapeutic branches of interest focused on early and correct detection, as well as the best therapeutic approach. Radiology never ceases to develop newer technologies in order to give patients a clear, safe, early, and precise diagnosis; furthermore, in the last few years diagnostic imaging panoramas have been extended to the field of artificial intelligence (AI) and machine learning. On the other hand, clinical and laboratory tests, like flow cytometry and the techniques found in the “omics” sciences, aim to detect microscopic elements, like extracellular vesicles, with the highest specificity and sensibility for disease detection. If these scientific branches started to cooperate, playing a conjugated role in pathology diagnosis, what could be the results? Our review seeks to give a quick overview of recent state of the art research which investigates correlations between extracellular vesicles and the known radiological features useful for diagnosis.

## 1. Introduction

**Figure 1:**
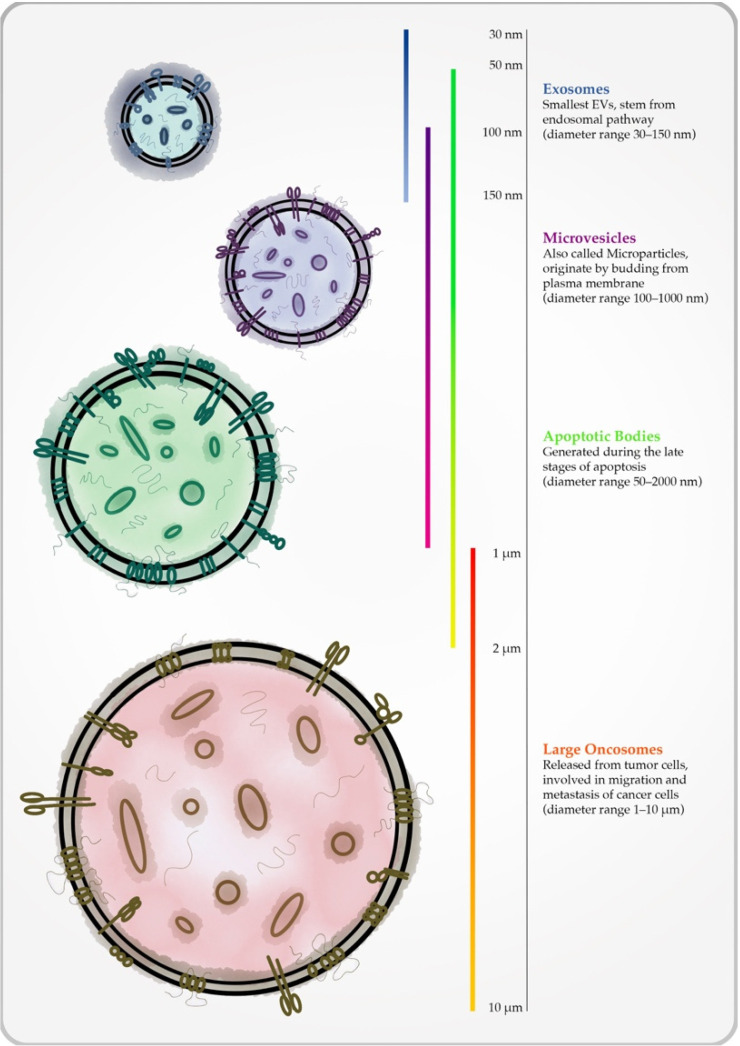
Schematic illustration of different typologies of extracellular vesicles (EVs).

Extracellular vesicles (EVs) are nanosized bilayer particles secreted by all cell types. Initially, their secretion was regarded as a mechanism for removing waste from the cells; however, it is currently known that EVs are key players in the biomolecular exchanges among cells, mediating intercellular cross-talk [1,2]. Therefore, they are involved in all pathophysiological processes, such as homeostasis, cell growth and differentiation, immune response, and many others [3,4]. Because of their involvement in these mechanisms, EVs are detectable in all biofluids, including milk, blood, urine, and amniotic fluid; therefore, they are perfect candidates for new biomarkers [1,5]. They are composed of a stable membrane-bound structure, which is linked to the biological stability of the EV cargo that protects the EV integrity from extracellular enzymes [6]. The cargo could be composed of proteins, lipid mediators, DNA molecules, RNAs, and microRNAs, and the quality and quantity of the EV loading depends on the trigger release and the cells’ bilayers [7,8]. The EV population includes three types of vesicles, named exosomes, microvesicles (MVs, also known as microparticles, MPs), and apoptotic bodies [9]. The classification of these three different populations is based on their biogenesis mechanisms, as well as on their sizes, properties, and roles in pathophysiological conditions. Recently, The International Society of Extracellular Vesicles (ISEV) proposed a re-classification of EVs based on their relative size, identifying small or medium/large EVs, ranging between <200 nm and >200 nm, respectively (Figure 1) [9]; *Exocarta* [10], *Evpedia* [11], and *Vesiculepedia* [12] are three different databases which are useful for finding updated information on EVs [10,12].

The smallest EV subtype is represented by exosomes, which have diameters in the range between 30 and 150 nm [13,14]. Exosomes stem from the endosomal pathway, specifically from multivesicular body (MVB) membranes undergoing an invagination process, which induces the intraluminal vesicle (ILV) formation. ILVs become “exosomes” when they are secreted in the extracellular environment after the fusion of MVBs with the plasma membrane [15]. The composition of the exosomes, in terms of protein and lipid content, reflects their origin. A large number of molecules have been identified in exosomes from different cell origins. Of note, exosomes are highly enriched in tetraspanins (CD9, CD63, CD81, and CD82) that play a pivotal role in cell penetration, invasion, and fusion. Exosomes also express heat shock proteins (Hsp60, Hsp70, and Hsp90) that are involved in stress response, as well as in antigen binding/presentation. Some proteins, such as Alix and TSG101, which are MVB formation molecules, are involved in exosome release, while others such as annexins and Rab are responsible for membrane transport and fusion. Alix, flotillin, and TSG101 also participate in exosome biogenesis [16,17]. Exosomes also contain different RNAs and miRNAs specifically packaged by their parental cells. In addition to proteins and nucleic acids, the exosome lipid components also contribute to their bioactivity. Exosomes are, in fact, enriched in phosphatidylserine (PS), phosphatidic acid, cholesterol, sphingomyelin (SM), arachidonic acid, and other fatty acids, prostaglandins, leukotrienes, and functional lipolytic enzymes [1,18].

Microvesicles (MVs), also known as microparticles (MPs), present a diameter ranging from 100 to 1000 nm [19]. They originate by budding from the plasma membrane through mechanisms involving Rho-associated protein kinase (ROCK), which allows the formation of an actin complex [20]. MVs are theoretically secreted by all cell types, acting as carriers of biomolecules, such as enzymes, and are involved in glucose and amino acid metabolism and mitochondria-derived vesicles [21,22]. For these reasons, MVs express the phenotype of their parental cells [23,24,25].

Recently, a new population of large EVs, named large oncosomes, has been discovered [26]. They are large microvesicles (1–10 μm in diameter) released from tumor cells, and are able to deliver their cargo for long distances [27]. It has been demonstrated that large oncosomes are involved in the process that leads to the migration and metastasis of cancer cells [28].

Apoptotic bodies are membrane vesicles of 50–2000 nm, generated during the late stages of apoptosis [29]. It is now clear that apoptotic body genesis is the result of cell disassembly, which is a complex process that involves a number of highly coordinated morphological steps. Each cell type carries a specific mechanism of cell disassembly that generates different types and numbers of apoptotic bodies. Once released in the extracellular space, apoptotic bodies are phagocytosed by macrophages, parenchymal cells, or neoplastic cells and then they are degraded within phagolysosomes. In any case, apoptotic bodies express phosphatidylserine (an “eat me” signal) on their surface [30] and are enriched in caspases-3 and -7, as well as in ROCK1 and PANX1. Because of their origin, apoptotic body production is considered a hallmark of the apoptotic process. As with the other EV subtypes, apoptotic bodies deliver proteins, lipids, DNA molecules, and large amount of RNA [31,32].

EVs were proposed as highly promising biomarkers for the diagnosis and the monitoring of human diseases in different clinical settings [33]. They have enormous potential to cross biological barriers, therefore reflecting in the biofluids the pathophysiology of the different body compartments [34]. For these reasons, EVs have been proposed as a potential source for liquid biopsies in a tailored medicine context. In this scenario, new methodological approaches are emerging as highly promising tools for EV analyses [9]. Interestingly, new rapid and sensitive techniques based on combined flow cytometry/proteomics methods have been recently proposed for translating EV research into clinical practice [35,36]. These new approaches are also highly promising in the context of the study of the EV cargo in terms of RNA and miRNA molecules. The possibility to obtain pure EVs or EV subtypes by fluorescence activated cell sorting may open new routes in the deep sequencing of the EV-associated RNAs [37].

Based on this evidence, several authors looked for a rendezvous between the consolidated diagnostic practice, which uses different radiological methods, and liquid biopsies through extracellular vesicles, for true information-bearing elements about pathologies. Radiological imaging, in particular ultrasonography (US), computed tomography (CT), and magnetic resonance (MR), with or without the use of their relative contrast medium, is currently a crucial but sometimes insufficient tool in the early detection and easy diagnosis of different pathologies. The combinatorial information given by EVs and radiological imaging could improve diagnosis accuracy, leading to an earlier diagnosis of diseases, especially cancer. This translational research branch, the aim of which is not only to diagnose but to prevent diseases, could be defined with a neologism: radiovesicolomics. Radiovesicolomics could be considered a discipline that, through utilizing radiological and flow cytometry data sources, aims to create models for data integration and prediction to evaluate the complex functioning of various pathologies. The main aim of radiovesicolomics is to bring the struggle against diseases to the next level; earlier, more specific, and sensitive diagnosis in future could lead not only to a rapid therapeutic approach, but also to prevention before the pathology manifests itself.

With the present review we want to give a quick overview of the state of the art research studies found in literature which have the purpose to underline any correlation among radiological findings, flow cytometry, and the “omics” data of different diseases in the EV field (Table 1).

## 2. Cardiovascular Radiology & EVs

The roles of EVs in cardiovascular and cardiometabolic diseases ranging from genetic, acute, and/or chronic disorders (e.g., dilated cardiomyopathy, myocardial infarction, and heart failure, etc.) are well-known [38]. It is understood that apoptotic endothelial-derived MVs (EMVs) such as CD144^+^ can be a predictor of, and correlate with, coronary artery disease (CAD), and are considered to be a promising biomarker of thromboembolic conditions [39,40]. A clear link between EVs and cardiovascular radiology is currently being studied and little evidence is currently available. Chiva-Blanch et al. hypothesized a direct link between circulating MVs and coronary artery plaques identified by coronary computed tomography angiography in asymptomatic patients with familial hypercholesterolemia [41]. Given that Agatston et al. defined coronary artery calcium (CAC) as a plaque with an area of at least 1.03 mm^2^ and with an attenuation threshold of 130 Hounsfield units, it is worth remembering that CAC scoring is a non-invasive and consistent tool in depicting coronary artery atherosclerosis using computed tomography (CT), and that it is an independent prognostic marker for CAD [42,43]. The presence of coronary artery plaques was associated with elevated quantities of total annexin V (AV^+^), MVs, and MVs derived from granulocytes (CD66^+^/AV^+^), platelets (CD41a^+^/AV^+^, CD31^+^/AV^+^, CD41a^+^/CD31^+^/AV^+^), endothelial cells (CD62E^+^/AV^+^/^−^), and neutrophils (CD11b^+^/CD66^+^/AV^+^). In particular, the granulocyte-derived MVs correlated with the calcification burden of coronary atherosclerosis [41]. Miller et al. investigated the possibility that MVs could relate to CAC, by using CT in old women with a history of pre-eclampsia; the assumption was that hypertensive pregnancy disorders increased the risk of coronary atherosclerosis in postmenopausal women [44,45]. They found that MVs positive for vascular cell adhesion molecule-1 (ICAM-1) correlated with CACS in women with histories of pre-eclampsia, and that MVs derived from smooth muscle cells correlated with CACS only in women with histories of normotensive pregnancy. Several studies have pursued the aim of providing a coronary atherosclerosis estimation and an early biomarker of myocardial ischemia and infarction. High levels of EMVs are associated with acute coronary syndromes [46] indicate the presence of atherosclerotic plaques in the left anterior descending artery, rather than in other coronary arteries [47]. Jung et al. attempted to evaluate circulating EMVs and platelet-derived MVs (PMVs) as being predictors of the infarct size and the risk of ischemic myocardium in patients with ST-elevation myocardial infarction (STEMI) assessed by cardiac magnetic resonance [48]. The numbers of circulating CD31^+^/CD42^+^ PMVs and CD31^+^/CD42^−^ EMVs correlated with the area at risk of myocardium and troponin T values but not with infarct size. Instead, Kandiyil et al. studied the correlations between PMVs, EMVs, and ischemic stroke by evaluating the micro-embolic signals with transcranial Doppler ultrasound and the cerebrovascular ischemic events identified by diffusion magnetic resonance imaging [49]. Both PMVs and EMVs were associated with symptomatic stroke and positive diffusion-weighted imaging sequences, but only PMVs showed a connection with micro-embolic signals as a potential predictor of thromboembolic activity. Little data that links EVs to cardiovascular radiology are available to date, and further studies are required in order to identify peripheral blood EVs as a source of liquid biopsies in cardiovascular diseases.

## 3. Abdomen Radiology & EVs

Multimodality imaging and liquid biopsies can be used together to detect abdominal neoplastic diseases and predict their response to treatment [50,51]. A prospective study was conducted by Kassam et al. to see whether the combination of imaging and circulating biomarkers could predict treatment response in patients with rectal cancer [52]. Before and after chemo–radiant treatment, patients in the Kassam et al. study underwent positron emission tomography–magnetic resonance imaging (PET-MRI), CT-perfusion (CT-P), and liquid biopsies. While MRI is widely used in rectal cancer staging and treatment response evaluation, its efficacy in assessing neoadjuvant therapy response is limited by interobserver variability and overstating [53,54]. The ability of CT-P to combine anatomical and blood supply information is its advantage [55]. In the diagnosis and staging of colorectal cancer, the use of a liquid biopsy represents a modern concept. The latest research suggests that it may be able to detect residual tumor tissue after treatment, tumor relapse, and micrometastatic disease [56]. Kassam et al.’s study analyzed circulating tumor cells (CTCs), microparticles (MPs), and cell fragments as markers of treatment response together with multimodality imaging. CT-P alone was insufficient to identify treatment response and MRI did not provide significant information in the evaluation of the disease stage. However, valuable information was provided by the combination of CT-P and blood markers (CTCs, MPs, and fragments of cells) regarding anatomical and functional dysregulated vascularization and response to treatments. Endothelial transfer constant, permeability-surface area product, and mean transit time are tumor permeability indicators that were associated with blood biomarker levels [50].

Because of its silent progression, pancreatic cancer (PC) diagnosis results in a clinical challenge. Its symptoms, in fact, often emerge after the locoregional invasion has already begun. Early PC diagnosis using imaging techniques (CT, CEUS) is exceedingly rare as a result of this latency, and the prognosis is often poor [57,58]. In PC detection and follow-up, blood biomarkers, such as CA19-9, are regularly examined; however, even if those markers are highly sensitive and specific, PC diagnosis remains challenging [59]. The analysis of circulating extracellular vesicles may open new routes in the context of accessible and accurate biomarker identification [60,61]. Therefore, extracellular vesicles represent highly promising candidates for liquid biopsy assessments in the early diagnosis of PC, even if further studies are needed to implement the use of these biomarkers in combination with multimodality imaging techniques in the oncological field.

To our knowledge, there is a paucity of literature on studies that attempt to compare imaging techniques and circulating EV levels in order to enhance the diagnostic process and prognostic evaluation in non-neoplastic abdominal diseases. Some murine models were used to investigate the relationship between blood EVs and histologic/imaging features. Li et al., for example, studied cell-type-specific EVs in mice with nonalcoholic fatty liver disease (NAFLD). They noted that hepatocyte-, macrophage-, and neutrophil-derived EVs were significantly elevated in both male and female mice with induced NAFLD, and that they positively correlated with the nonalcoholic fatty liver disease activity score (NAS), as determined by histologic and MRI parameters [62]. Despite the enormous potential of liquid biopsies, no research has been undertaken to extend the spectrum of non-neoplastic diseases, the detection of which could benefit from the use of circulating biomarkers conjugated with imaging techniques. Acute and chronic pancreatitis, as well as infectious/inflammatory bowel diseases, are just a few examples of those. Recent studies on EV testing in these pathologies have shown promising results. However, in order for detection to be more reliable, they must be correlated with conventional diagnostic instruments [61,63].

## 4. Chest Radiology & EVs

EVs have been implicated in the pathogenesis of different pulmonary pathologies. The role of EVs in inflammation processes, including lung inflammation, is largely recognized [64,65,66]. It has been demonstrated that the coagulation cascade is related to pulmonary fibrosis (PF) pathogenesis. Type II pneumocytes from patients with pulmonary fibrosis (PF) secondary to systemic sclerosis, as well as with idiopathic PF (IPF), expressed upregulated levels of tissue factor (TF) [67]. It is also known that locally synthesized coagulation factor X contributes to the fibrotic evolution of lung injury [68]. Therefore, the procoagulant role of TF-bearing EVs in PF pathogenesis was analyzed, and the resulting EV-associated TF activity increased in PF patients compared to healthy subjects, and was also related to the disease severity [69]. PF patients were then grouped as IPF and non-IPF on the basis of their CT features, suggestive of usual interstitial pneumonia, demonstrating that the EV-associated TF activity was higher in IPF than in non-IPF patients [70].

Many studies explored the role of alveolar endothelial cell apoptosis in the pathogenesis of the chronic obstructive pulmonary disease and emphysema, focusing on EVs of endothelial origin expressing CD31 (platelet-endothelial cell adhesion marker 1) [71,72]. Thomashow et al. analyzed the relationship of circulating EV levels with COPD, pulmonary microvascular blood flow assessed by MRI, diffusing capacity of carbon monoxide (DLCO), and hyperinflation [73]. This study included 180 participants that underwent spirometry, CT scan, gadolinium-enhanced MRI, diffusing capacity, and plethysmography. It was demonstrated that CD31^+^ EVs were increased in COPD patients compared to control subjects; higher levels of CD31^+^ EVs were also associated with the percentage of emphysema on CT scan, reduced PMBF, and lower DLCO.

Interestingly, it has been demonstrated that cancer-derived EVs carry miRNAs involved in the recruitment and reprogramming of the tumor environment components [74]. Given that lung cancer has very low survival rates (lower than 5 years) [75], and has high mortality rates due to advanced stages diagnosis, EV miRNAs have been underlined as ideal non-invasive biomarkers for early diagnosis and as a diagnostic/prognostic, as well as being predictive tools in the lung cancer context. In this scenario, it is known that the epidermal growth factor receptor (EGFR) overexpression correlates with poor prognosis in many types of malignancies, including non-small cell lung cancer (NSCLC). The majority of EGFR-mutant NSCLC tumors that show an initial radiological response to EGFR tyrosine kinase inhibitors develop different mechanisms of resistance [76]. The possibility to determine the presence of EGFR mutations in EVs may substitute invasive procedures for the diagnosis, or the follow-up, of cancer patients, reducing the complications derived from tumor biopsies, and anticipating progression. Taverna et al. reported the case of a 70 year old woman diagnosed with stage IV NSCLC and harboring an EGFR activating mutation [77]. The patient was treated with Gefitinib 2, and after two months of treatment a CT scan showed a partial response in the primary tumor. Before the beginning of the treatment with Gefitinib, peripheral blood was collected, and the analysis confirmed the downregulation of miR-122 in the EVs. This clinical case underlined that NSCLC EVs and the related miRNAs might represent new promising biomarkers. Moreover, it was demonstrated in NSCLC patients that low blood concentration of circulating endothelial-derived EVs before treatment was strongly associated with longer overall survival rates and higher disease control rates in patients treated with immune checkpoint inhibitors [35].

It is also known that most lung cancers are first diagnosed by chest imaging as lung nodules. In this context, the identification of noninvasive approaches for the early diagnosis of lung cancer remains one of the major challenges. Recently, the study of nodule features, together with the identification of specific clinical risk factors, have been applied to predict malignancy [78,79]. This method was based on nodule size measurements and the time-monitoring of nodule size increase through imaging techniques [80,81]. It has also been demonstrated that benign nodules show different protein patterns in respect to lung cancer [82].

To the best of our knowledge, there are no recent studies about the correlation of acute distress respiratory syndrome (ARDS), EVs, and diagnostic tools.

## 5. Neuroradiology & EVs

Several studies that tried to correlate EVs with neurological disorders (neurodegenerative changes, inflammatory, and cerebrovascular diseases) have been conducted to date. Neurons and astrocytes, like other human cells, may produce EVs containing mRNAs, miRNAs, proteins, and lipids that are released in the extracellular space and may be considered as promising biomarkers in different neurological conditions [83,84]. EVs are also able to cross the blood–brain barrier [85]. Geraci et al. demonstrated the relationship between EVs levels in the cerebrospinal fluid (CSF) and the severity of multiple sclerosis (MS) identified by MRI [86]. The authors showed that CSF-EV concentration directly correlated with progressive MS and with a relapsing clinical phase. Besides this, CD19^+^/CD200^+^ CSF-EVs were decreased during the clinical and radiological active phase of relapsing MVs in contrast with IB4^+^ CSF-EVs that were elevated in the stable form. The presence of gadolinium-positive lesions was associated with a statistically significant elevation of CD4^+^/CCR5^+^. Elahi et al. investigated the possibility that endothelial-derived exosomes were related to white matter hyperintensities (WMH) on fluid-attenuated inversion recovery (FLAIR) and/or T2-weighted imaging [87]. This results from the assumption that WMH pathogenesis derives from blood–brain barrier dysfunction and endothelial pathologies [88,89]. Despite the limited population of the study, the authors found that the cargo protein levels of endothelial-derived exosomes (LAT1, GLUT1, NOSTRIN, and P-GP) were significantly higher in asymptomatic patients with WMH, rather than subjects without WMH. This finding could represent a future early biomarker in the asymptomatic stage of inflammatory and neurodegenerative disorders and a promising tool for targeted therapies. Furthermore, Kanhai et al. evidenced a high level of EV-CD14^+^ and EV-Cystatin C in patients with WMH and brain parenchymal atrophy evaluated with MRI, in contrast to EV-Serpin G1 and EV-Serpin F2 [90]. It is known that PMVs are considered as a marker of thromboembolic diseases due to their fundamental role in blood coagulation [91]. Kuriyama et al. hypothesized that high PMV levels were correlated with cerebrovascular disorders (cerebral infarctions) by performing MRI, magnetic resonance angiography, and carotid ultrasound [92]. Acute atherosclerotic strokes revealed elevated PMV serum levels as opposed to cardiogenic strokes; furthermore, their elevation was directly connected with cervical atherosclerosis, as defined as an intima–media thickness (IMT) >1.1 mm. Some authors studied the link between EVs and Alzheimer’s disease by comparing EVs values with hippocampal volume as an indicator of neuronal injury. Picciolini et al. found a partial correlation between a specific neural EV population (microglia/macrophages—IB4, CD11b) identified by surface plasmon resonance imaging (SPRi) technology and total hippocampal volume detected by MRI analysis [93]. In particular, the SPRi intensity CD11b/IB4 ratio could be considered as a potential new biomarker of severity, progression, and treatment response in patients with Alzheimer’s disease. Wang et al. hypothesized an association between plasma exosomes and the atrophy of the entorhinal cortex and hippocampus determined with MRI [94]. They found that higher exosomal β-site amyloid precursor protein cleaving enzyme-1-antisense transcript (BACE1-AS) levels are inversely proportional to the volume and thickness of the right entorhinal cortex in Alzheimer’s disease. However, no statistically significant difference in the hippocampal volume has been proven between patients with Alzheimer’s disease and the control group, maybe due to the limited number of the enrolled patients. Furthermore, through the assumption that pSer312-IRS-1 and p-panTyr-IRS-1 are fundamental in Alzheimer’s disease pathogenesis [95], Mullins et al. demonstrated that plasma exosomes enriched for neural origin (L1CAM) correlate with the brain atrophy evaluated with T1-weighted magnetization-prepared rapid gradient-echo (MPRAGE) images [96]. In detail, pSer312-IRS-1 levels were associated with greater brain atrophy rather than p-panTyr-IRS-1 levels, as a result of their deteriorating and protective role in Alzheimer’s disease, respectively.

## 6. EVs Targeted Contrast Media in Current Imaging

Based on tumor type or specific disease features, MVs could be engineered with crystalline ion or non-ion markers to obtain highly biocompatible targeted contrast agents [97,98]. Currently, superparamagnetic iron oxide nanoparticles (SPIOn), ultrasmall superparamagnetic iron oxide nanoparticles (USPIOn), and gadolinium represent the best diagnostic tools for the MRI detection of marked EVs [97,98]. Alternatively, gold rearranged EVs constitute the equivalent media in CT [99,100]. Several investigations have demonstrated the potential efficacy of labeled EVs as contrast media for diagnostic purposes [97,100]. No investigations on human beings are currently available. Rayamajhi et al. developed a gadolinium-hybrid EV contrast medium which does not significantly modify magnetic properties and relaxation times compared to classic gadolinium-based intravenous contrasts [101]; furthermore, this type of contrast medium demonstrated an increased tumor uptake and a reduced diffusion to the extracellular compartment in osteosarcoma tumor-bearing mice. The targeted drug showed promising results due to specific cancer gadolinium collection and lower gadolinium plasma concentration compared to common gadolinium-based agents. Despite features which could reduce drug toxicity and increase lesion conspicuity during cross-sectional imaging protocols, it should be noted that the very different half-life between EVs and contrast media composed of magnetic nanoparticles could generate signal persistence even in absence of carriers. Literature data revealed high EV tissue concentration for up to 24 h, while labeled iron nanoparticles showed hepatic clearance for about 3–4 days based on specific nanoparticles, justifying potential tissue magnetic nanoparticle accumulation [102,103].

Tumor derived EVs fused with gold iron oxide nanoparticles, similar to gadolinium- coated EVs, exhibited significant selective uptake in murine breast cancer cells during MRI studies compared to other tissues; therefore, this achievement, linked to the possibility of the chemical combination of gold-coated EVs with antiblastic drugs, could be a promising development in cancer treatment strategies, being simultaneously timesaving and highly selective in diagnostic and therapeutic approaches [104]. Further investigations suggest that small numbers of adipose stem cells can be detected with MRI following the administration of ultra-small super paramagnetic iron oxide (USPIO) engineered with specific exosomes. Busato et al. demonstrated that MRI was able to detect adipose stem cell-USPIO exosomes in murine models, revealed as T2* signal selective hypointensity [105]; these findings could be a milestone for the application of MR labeled exosome tracking in neuropathology, in which adipose stem cells seem to be widely involved. Similar in vivo investigations display the effectiveness of MR tracking SPIOn-wrapped melanoma-derived exosomes in popliteal lymph nodes of murine models. Selective homing of USPIO melanoma exosomes could be a perspective tool in the identification of residual disease after the surgical eradication of melanoma, or in early detection of small melanoma metastases [106]. Mesenchymal stem cell gold nanoparticle-labeled exosomes demonstrated selective uptake in neurodevelopmental disorders, ischaemic stroke, and other brain diseases via GLUT1 transporters during CT imaging in animal models [100], providing support to the targeted diagnosis of neurodegenerative disease. Gold nanoparticles combined with melanoma-derived exosomes preferentially accumulate in neoplastic cells, even if there is still uncertainty about the possibility of identifying distant melanoma localization in murine models [99]. Moreover, potential limitations of exosome-based contrast media are represented by a lack of standardization in exosome manipulation and the absence of biosafety profiles [99,101].

## 7. Artificial Intelligence, Radiomics & EVs

The advent of artificial intelligence (AI), followed by its medical applications, has improved health outcomes given that it integrates human intelligence, maximizing the diagnostic and prognostic value of actual tests and minimizing the medical burden [107].

The availability of large datasets, together with the significant advances in radiomic methods of analysis and machine learning (ML) approaches, means the possibility of applying diagnostic radiological imaging, creating an optimal platform to connect clinical medicine with AI. The fact that CT and MR are not only images but also data, allowed the emergence of “radiomics” [108]. In detail, gray scale images traditionally obtained by radiological applications can be visualized as data following the application of complex algorithms, allowing the identification and characterization of different features and patterns of detection normally invisible to the naked eye [109].

It must be specified that the term AI refers to the ability of ML to perform different tasks associated to human intelligence abilities (i.e., problem-solving and pattern recognition). Recently, this concept has benefited from a computing power increase and the possibility of using large datasets to train these systems. In the biomedical field, the term “big data” is used to describe large datasets, including data coming from genetics, proteomics, metabolomics, and miRNomics obtained from large biobanks or large cohorts of patients. Even if ML algorithms can be trained using small as well as large datasets, the possibility to use large numbers of data allows us to obtain a sample variation useful for maximizing the external and internal validity of the trained algorithms (reproducibility). Furthermore, the possibility of applying the analysis to large datasets also reduces the overfitting risk [110].

Two categories of learning exist in the ML field: unsupervised and supervised. The operator, on the basis of his experience, the dataset nature, and the study purpose, selects the right model in each setting. In this context, “radiomics” refers to the application of complex algorithms to radiological images, allowing the calculation of a large number of parameters related to the shape, attenuation, and “consistency” of a given area of interest.

In this way, the methods of radiomics bridge the gap between scanning and the generated datasets that are used by ML to generate AI systems. This approach was recently proposed in oncology with promising results [111,112,113]. The biophysical parameters of EVs can be investigated with ML. For example, in 2003, Won et al. used AI analysis to differentiate renal cell carcinoma (RCC) patients from healthy subjects and others with urological diseases, based on five protein biomarkers of serum [114]. Moreover, Zheng et al. predicted the early stage RCC patients using a biomarker cluster that was identified by the serum metabolomics method and ML algorithms [115]. EV data can be further integrated with other “omics” disciplines, including radiomics, gene expression, protein expression, or metabolites. For example, Chen et al. profiled four surface biomarkers including HER2, GPC-1, EpCAM, and EGFR for serum-derived EVs through DNA points accumulation for imaging in the nanoscale topography (DNA-PAINT) technique. In their results, the authors accurately differentiated pancreatic cancer and breast cancer from unknown samples [116]. It is reasonable to assume that the integration of EVs and other “omics” disciplines such as radiomics, proteomics, genomics, metabolomics, and transcriptomics will provide in the future new opportunities for novel target identification and validation in the field of cancer diagnosis, cancer progression, and EV-based anticancer therapies which aim to a personalized medicine.

## 8. Conclusions

The presented scenario suggests that EVs could be considered as a clear and specific biomarker able to support the radiological features and data; furthermore, the increasingly strong potential of the use of EVs as liquid biopsies seems to have real benefits, even with radiology. The conjugated approach of these two diagnostic paths, considering also the emerging role of radiomics in the radiological panorama, could lead towards the development of the aforementioned new medical research branch, radiovesicolomics.

## Figures and Tables

**Table 1:** Summary table of the major topics explained in the subsequent paragraphs.

Chapter	Topic	Main Authors & Research Works
*Cardiovascular Radiology & EVs*	Studies collection on the correlation between microvesicles, peripheral blood values and coronary artery calcification, ST-elevation, myocardial infarction, and ischemic stroke evaluated through multimodal imaging.	Chiva-Blanch, G. et al. Liquid Biopsy of Extracellular Microvesicles Maps Coronary Calcification and Atherosclerotic Plaque in Asymptomatic Patients with Familial Hypercholesterolemia. *Arterioscler. Thromb. Vasc. Biol.* **2019**, 39, 945–955, doi:10.1161/ATVBAHA.118.312414.
*Abdomen Radiology & EVs*	The uses of liquid biopsies used together with multimodal imaging for rectal cancer treatment response, early diagnosis of pancreatic cancer, and in the diagnosis and prognosis of non-alcoholic fatty liver disease.	Kassam, Z et al. A prospective feasibility study evaluating the role of multimodality imaging and liquid biopsy for response assessment in locally advanced rectal carcinoma. *Abdom. Radiol. (New York)* **2019**, 44, 3641–3651, doi:10.1007/s0026-02135-8.
*Chest Radiology & EVs*	Role of extracellular vesicles in pulmonary fibrosis, chronic obstructive pulmonary disease, emphysema, and lung cancer.	Imokawa, S et al. Tissue factor expression and fibrin deposition in the lungs of patients with idiopathic pulmonary fibrosis and systemic sclerosis. *Am. J. Respir. Crit. Care Med.* **1997**, 156, 631–6, doi:10.1164/ajrccm.156.2.9608094.
*Neuroradiology & EVs*	Link between extracellular vesicles and MR imaging of multiple sclerosis, white matter hyperintensities, stroke, Alzheimer’s disease, and cortex atrophy.	Picciolini, S. et al. An SPRi-based biosensor pilot study: Analysis of multiple circulating extracellular vesicles and hippocampal volume in Alzheimer’s disease. *J. Pharm. Biomed. Anal.* **2021**, 192, 113649, doi:10.1016/j.jpba.2020.113649
*EVs targeted contrast media in current imaging*	Extracellular vesicle manipulation to obtain highly biocompatible targeted contrast agents.	Lorenc, T. et al. Current Perspectives on Clinical Use of Exosomes as a Personalized Contrast Media and Theranostics. *Cancers* **2020**, 12, doi:10.3390/cancers12113386.
*Artificial Intelligence, Radiomics & EVs*	Personalized medicine as the aim of integration between complex algorithms, extracellular vesicles, and other “omics” disciplines.	Lambin P et al. Radiomics: the bridge between medical imaging and personalized medicine. *Nat. Rev. Clin. Oncol.* **2017**, 14, 749–762, doi:10.1038/nrclinonc.2017.141.

## Data Availability

Not applicable.
